# Hepatobiliary Actinomycosis, a Rare Presentation of a Rare Disease!

**DOI:** 10.7759/cureus.12413

**Published:** 2020-12-31

**Authors:** Iayla Fatima, Frederick Pretorius, Stefan Botes, Rachel Swanwick

**Affiliations:** 1 General Surgery, St. Luke’s General Hospital, Kilkenny, IRL; 2 Surgery, St. Luke's General Hospital, Kilkenny, IRL

**Keywords:** actinomycosis, actinomyces

## Abstract

Actinomycosis is a chronic granulomatous infection caused by opportunistic bacteria, Actinomyces. These bacteria lack virulence and cause disease when there is a breach in the integrity of the mucosa. Diagnosis of Actinomycosis is challenging and less than 10% of abdominopelvic cases are diagnosed preoperatively. The treatment involves prolonged course of antibiotics with or without removal of tissue. In this case report we will present a case of hepatobiliary actinomycosis which was managed by a combination of both surgical and medical treatment.

## Introduction

Actinomyces are gram positive, filamentous, anaerobic to microaerophilic bacteria endogenous to the oral cavity, gastrointestinal and genitourinary tracts [1]. Actinomycosis is a subacute to chronic infection caused by these organisms and is characterized by granulomatous inflammatory reaction with the formation of multiple abscess and sinuses [2]. The incidence of infection with Actinomyces is 1 in 300,000 and risk factors include age (20-60 years), male gender, immunosuppression and local tissue damage [3]. Actinomycosis most frequently involves the face and the neck (50%) followed by abdomen (20%) and thorax (15%) [4]. Hepatobiliary actinomycosis is extremely rare and may present as biliary colic, acute or chronic cholecystitis or pancreatitis [5]. In this case report we present a case of hepatobiliary actinomycosis.

## Case presentation

A 47-year-old female, presented to the emergency department with right upper quadrant pain, vomiting and fever for two days. She was previously known to have gallstones and was awaiting cholecystectomy for recurrent biliary colic. On examination, she was found to be tender in right upper quadrant with minimal rebound tenderness. Her liver function tests on admission showed total bilirubin 12.4 umol/L (2.0-21 umol/L), alanine aminotransferase (ALT) 25 IU/L (5-33 IU/L), gamma-glutamyl transpeptidase (GGT) 44 IU/L (3-40 IU/L), alkaline phosphatase 78 IU/L (30-130 IU/L), WBC 7.7 x 10^9^ (4-10 x 10^9^) and C-reactive protein (CRP) 32 mg/L (0-5 mg/L). All other laboratory parameters were within normal limits. Clinical impression of acute cholecystitis was made and ultrasound was done.

**Figure 1:**
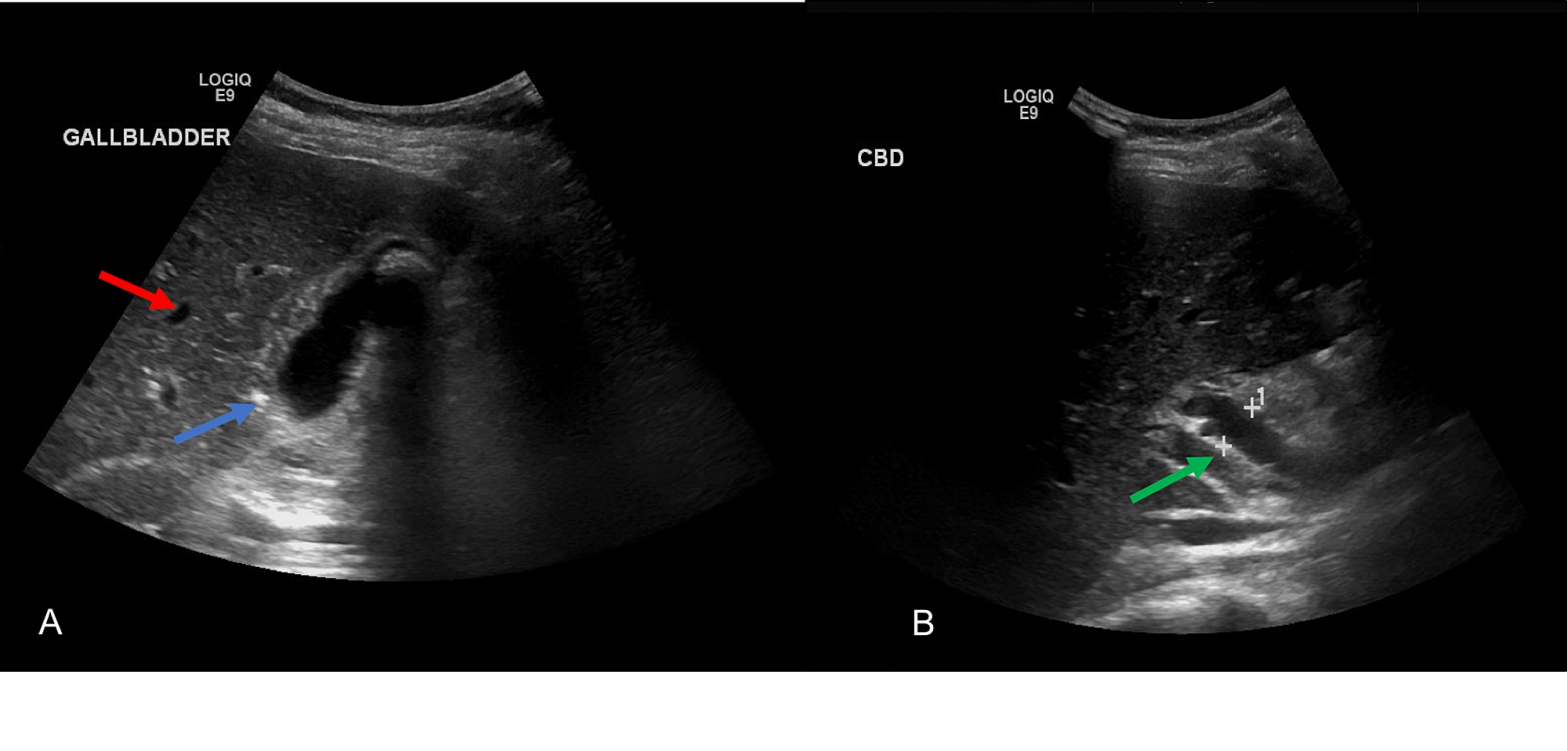
Ultrasound image of gallbladder (A) and common bile duct (B) shows intrahepatic duct dilation (red arrow), thickened gallbladder wall (blue arrow), dilated common bile duct with no filling defect (green arrow). Measurement of common bile duct is illustrated by '+'

Ultrasound revealed a thick-walled gallbladder, gallstones, dilated common bile duct (CBD) with intra and extrahepatic duct dilation; no stone was identified in the CBD (Figure 1A, 1B).

**Figure 2:**
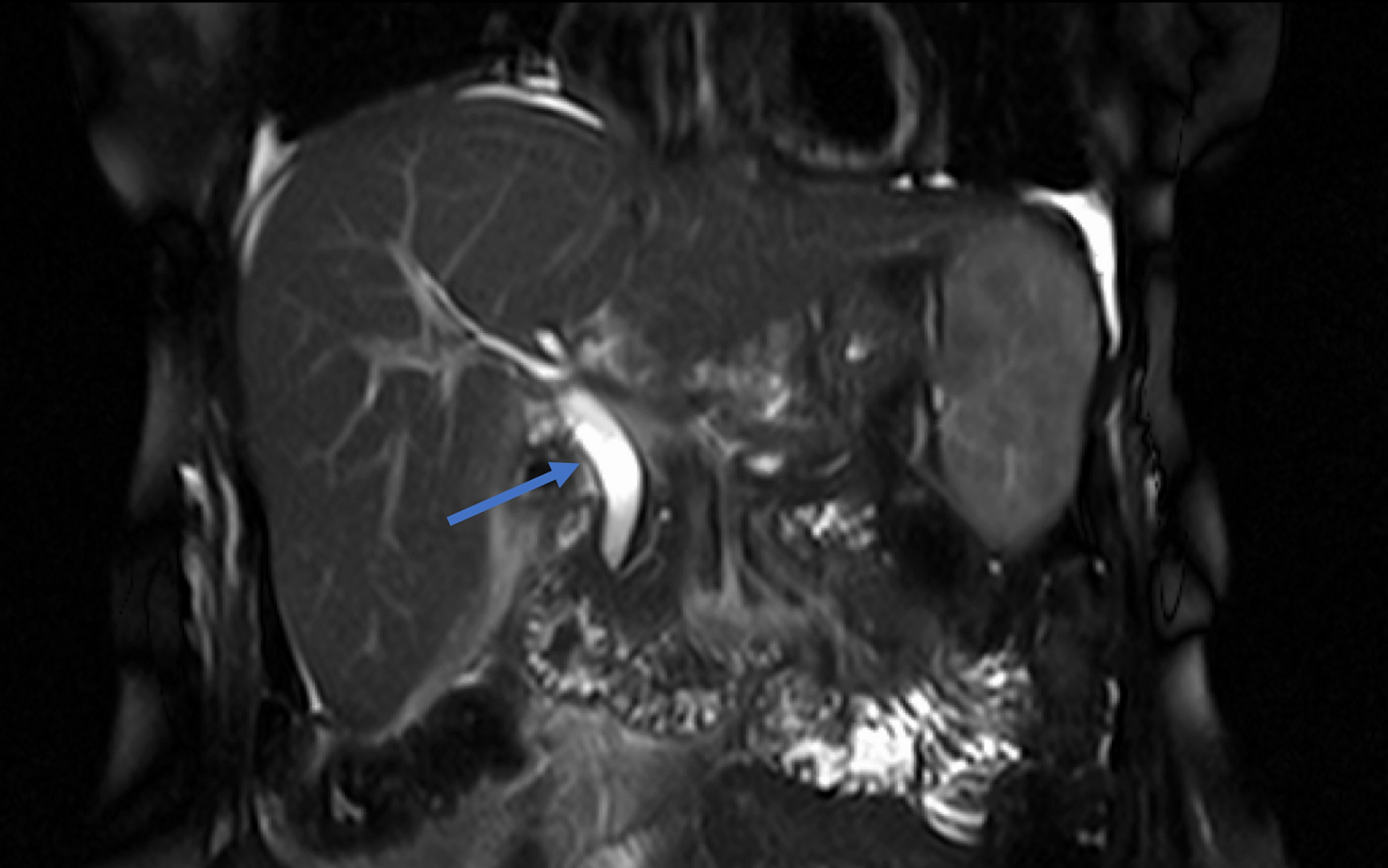
MRI showing dilated common bile duct with no filling defect (blue arrow).

Following this report a magnetic resonance imaging (MRI) was performed which confirmed a dilated common bile duct but no stones were identified in the common bile duct (Figure 2). The patient underwent a cholecystectomy.

Laparoscopic approach was undertaken and the omentum was found to be wrapped around the gallbladder, on mobilization of the omentum, a contained perforation of the gallbladder fundus was identified. Severe fibrosis was noted at the Hartmann’s pouch making it difficult to identify the cystic duct and artery, a dilated CBD was also noted intraoperatively. Due to difficult dissection, gallbladder was opened at the level of the Hartmann’s pouch, stones were removed and a subtotal cholecystectomy was done. A drain was placed in the gallbladder fossa. Apart from the histopathology of the gallbladder the peritoneal fluid (collected prior to gallbladder mobilisation) and bile aspirate were sent for gram stain and culture.

The patient had an uneventful postoperative course and the drain was removed on third postoperative day. However, the bile culture grew Actinomyces naeslundii and as per microbiologist’s suggestion the patient was started on Benzylpenicillin 2.4 gm IV four times a day for two weeks followed by three months of amoxicillin. The peritoneal fluid culture and the tissue culture from the abscess cavity showed no growth and the histopathology reported active chronic cholecystitis.

## Discussion

Actinomyces species encompasses more than 30 bacteria of which Actinomyces israelii is the most commonly identified in the clinical diagnosis of actinomycosis. Actinomyces are non-spore forming gram positive rods and except Actinomyces meyeri, which is short and non-branching, they all form branching filamentous rods [6]. Due to these large filaments lymphatic spread is rare [2]. The bacteria is considered opportunistic and lacks virulence causing symptoms in immunocompromised states or when there is a breach in normal defenses [1, 7, 8].

Primary diagnosis of actinomycosis is difficult and computed tomography (CT) in abdominal actinomycosis may show thickened bowel, pelvic or peritoneal mass with extensive infiltration [9]. Bacterial culture and pathology are the mainstay in diagnosis and identification of these bacteria from a sterile site is confirmatory. Surgery is undertaken for suspected neoplasm or to drain the abscess [3].

Histopathology may reveal sulfur granules; these are basophilic masses with eosinophilc terminals on hematoxylin and eosin staining but it's present in about 75% of cases [10]. Combined antibiotic and operative treatment is curative in 90% of the cases for actinomycosis [11]. Antibiotic treatment includes IV Penicillin G (18-24 million units)/day for 2-6 weeks followed by amoxicillin (500-750 mg) three to four times a day for 6-12 months [12].

A similar case was reported in 2005 of a 50-year-old Sudanese male, who presented with clinical features of acute cholecystitis and was initially managed conservatively and brought back for a delayed cholecystectomy after six weeks. Although we operated on our patient on the same admission the intraoperative findings in both cases were of dense adhesions and fibrosis. Both the patients recovered well postoperatively with prolonged course of antibiotics [13].

In our case of hepatobiliary actinomycosis the proposed mechanism is the retrograde spread from duodenum into the common bile duct, liver is involved in 5% of these patients. To the best of our knowledge so far only 30 cases have been reported of gallbladder involvement and two cases of isolated CBD involvement [4, 8, 14].

## Conclusions

Our case discussed hepatobiliary actinomycosis which is extremely rare. The article highlights the difficulty in diagnosis and challenges in surgical approach. Clinicians should be aware of the intraoperative findings so that appropriate cultures are ordered and tissue is carefully examined. Currently all reported cases of gallbladder actinomycosis are diagnosed after surgical resection and the role of endoscopic retrograde cholangiopancreatography (ERCP) is not well investigated in diagnosis. Theoretically, if actinomycosis is diagnosed through culture of bile sample (obtained through ERCP), and if patient's clinical condition allows, it may be treated with prolonged course of antibiotics only.
